# Very Early Diagnosis and Management of Congenital Erythropoietic Porphyria

**DOI:** 10.1177/00099228221128661

**Published:** 2022-10-11

**Authors:** Marie-Pier Desjardins, Lamia Naccache, Audrey Hébert, Isabelle Auger, Pierre Teira, Marie-Claude Pelland-Marcotte

**Affiliations:** 1 CHU de Québec, Centre Hospitalier de l’Université Laval, Department of Pediatrics, Université Laval, Québec City, QC, Canada; 2 CHU de Québec, Centre Hospitalier de l’Université Laval, Division of Dermatology, Department of Medicine, Université Laval, Québec City, QC, Canada; 3 CHU Sainte-Justine, Division of Hematology/Oncology, Department of Pediatrics, University of Montréal, Montréal, QC, Canada

**Keywords:** congenital erythropoietic porphyria, hematopoietic stem cell transplantation, hematology

## Abstract

Congenital erythropoietic porphyria (CEP), a rare form of porphyria, is caused by a defect in the heme biosynthesis pathway of the enzyme uroporphyrinogen III synthase (UROS). Uroporphyrinogen III synthase deficiency leads to an accumulation of nonphysiological porphyrins in bone marrow, red blood cells, skin, bones, teeth, and spleen. Consequently, the exposure to sunlight causes severe photosensitivity, long-term intravascular hemolysis, and eventually, irreversible mutilating deformities. Several supportive therapies such as strict sun avoidance, physical sunblocks, red blood cells transfusions, hydroxyurea, and splenectomy are commonly used in the management of CEP. Currently, the only available curative treatment of CEP is hematopoietic stem cell transplantation (HSCT). In this article, we present a young girl in which precocious genetic testing enabled early diagnosis and allowed curative treatment with HSCT for CEP at the age of 3 months of age, that is, the youngest reported case thus far.

## Introduction

Congenital erythropoietic porphyria (CEP) is a rare form of porphyria. This autosomal recessive inherited heme metabolism disorder is caused by a defect of the enzyme uroporphyrinogen III synthase (UROS), an enzyme in the heme biosynthesis pathway. Uroporphyrinogen III synthase deficiency leads to an accumulation of porphyrins in the erythroid precursors of bone marrow, red blood cells, and tissues such as skin, bones, teeth, and spleen.^1-3^ The exposure of nonphysiological accumulated porphyrins to sunlight causes oxidative stress leading to cell death.

More than 50 pathogenic mutations in UROS have been identified explaining the various clinical presentations of CEP ranging from severe antenatal manifestations causing hydrops fetalis to adult-onset mild cutaneous lesions.^2^ Congenital erythropoietic porphyria is characterized by severe skin photosensitivity and skin fragility, causing blisters, erosions, secondary skin infections, and later changes such as atrophy and mutilating scars. Accumulated porphyrins in the erythroid precursor of bone marrow and red blood cells result in ineffective erythropoiesis and long-term intravascular hemolysis. Other manifestations of CEP such as brown-red urine, erythrodontia, corneal ulceration, osteoporosis, and pathological bone fractures are corollaries of excessive porphyrins deposition in tissues. The diagnostic of CEP is based on the markedly elevated levels of porphyrins in urine, clinically observed with a dark-brown staining of the diapers. High levels of porphyrins in red blood cells and feces are also noted. Genetic testing identifying UROS mutation confirms the diagnosis.

Patients suffering from CEP have a poor outcome and quality of life.^4^ Several supportive therapies such as strict sun avoidance, physical sunblocks, red blood cells transfusions,^5^ hydroxyurea^6^ and splenectomy^7^ are commonly used in the management of CEP.^8^ Currently, the only available curative treatment of CEP is hematopoietic stem cell transplantation (HSCT). In this article, we present, to our knowledge, the youngest case of successful HSCT for CEP.

## Case Description

A baby girl was born through vaginal delivery at 38^5/7^ weeks of gestation, with severe symmetric intrauterine growth retardation (birth weight 2240 g, <3^e^ percentile). Parents had a consanguineous union (third-degree cousins). This was the fourth pregnancy of a multiparous female, who had first lost twins during pregnancy (twin A: death in utero at 8-9 weeks of gestation; twin B: died at birth with anhydramnios, splenomegaly, extramedullary hematopoiesis, erythroblastosis, and cholestasis with hemosiderosis) and later had a healthy girl. This pregnancy was complicated by intrauterine growth retardation and other ultrasound abnormalities such as cystic hygroma at 12 weeks and later development of short femur, cardiomegaly, and pericardial effusion suggesting hydrops fetalis. Antenatal investigation including amniocentesis, karyotype, comparative genomic hybridization (CGH), and infectious panel were noncontributory.

**Figure 1.**
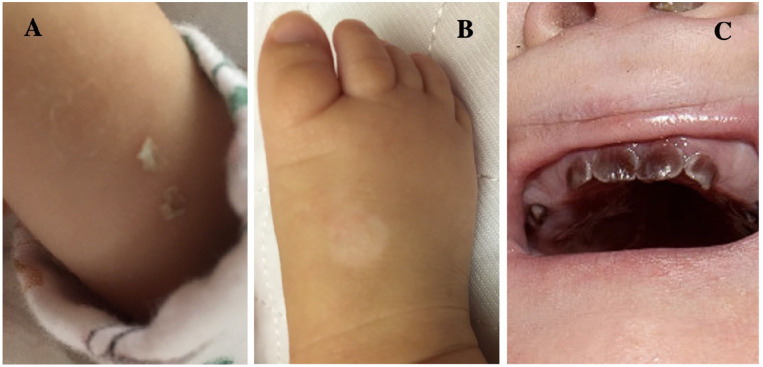
(A) Blue-red skin papules eruption, (B) cutaneous blister on the pulse oximetry site and (C) erythrodontia (pictures provided with the family’s authorization).

At birth, our patient presented with hepatosplenomegaly, hematuria, and blue-red skin papules eruption compatible with blueberry muffin rash (Figure 1A). She later developed a cutaneous blister on the pulse oximetry site (Figure 1B).

Laboratories showed regenerative normocytic anemia (hemoglobin: 95 g/dL, reticulocytes count: 10.7%), thrombocytopenia (platelet count: 84 × 10^9^/L) and cholestasis (total and direct bilirubin: 100 μmol/L and 65 µmol/L [8× the upper limit of normal], respectively) without associated transaminitis. The complementary work up, including blood smear, Coombs test, activity of pyruvate kinase and hexokinase, lactate dehydrogenase and infectious screening was negative.

**Figure 2.**
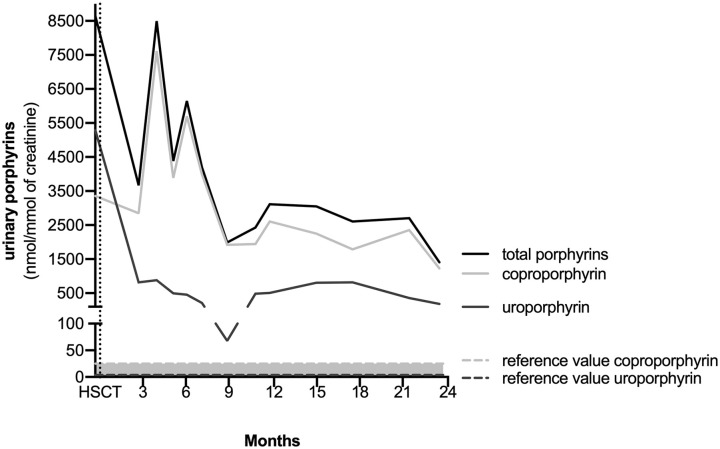
Levels of urinary porphyrins before and after HSCT in CEP. Abbreviations: HSCT, hematopoietic stem cell transplantation; CEP, congenital erythropoietic porphyria.

The family history prompted an early genetic referral and genetic testing. An extended metabolism gene panel (*Fulgent Genetics*) revealed a homozygous mutation in the UROS gene, variant (c.217>C, p.[Cys73Arg,]) leading to the early diagnosis of CEP. The homozygous c.217T>C [p. Cys73Arg] mutation, resulting in a less than 1% of normal UROS activity, is correlated with severe phenotype leading to blood transfusion dependency from birth.^9^ Urinalysis showed markedly elevated levels of uroporphyrin and coproporphyrin (Figure 2).

Supportive management included strict sun avoidance, physical sunblocks, and blood transfusions. However, regarding the extremely young onset of the disease and the poor expected outcome, an allogenic HSCT was offered. Therefore, at 3 months of age, the patient received an unrelated HSCT from a 10/10 HLA identical donor. The conditioning regimen was based on busulfan (days −6 to −33; 5 mg/kg/dose), fludarabine (days −8 to −3; 1 mg/kg/dose) and alemtuzumab (days −8 to −6; 0.167 mg/kg/dose). Cyclosporine and mycophenolate mofetil were administered as graft-versus-host disease (GVHD) prophylaxis. Anti-infectious prophylaxis included antibacterial prophylaxis with amoxicillin, antifungal prophylaxis with fluconazole, and micafungin, trimethoprim, palivizumab, and weekly intravenously administered immune globulins. The neutrophil engraftment was achieved on day +15. Full donor chimerism was confirmed 2 months after HSCT.

The HSCT was complicated by thrombotic microangiopathy (TMA) and recurrent pericardial effusion. The patient developed TMA approximately around day +7, manifesting with acute kidney failure, pericardial effusion, hypertension, hematuria, and proteinuria combined with markedly elevated C5b9 in blood. Thrombotic microangiopathy was successfully treated with changing cyclosporine to prednisone and eculizumab. Recurrent pericardial effusion occurred during prednisone tapering with no sign of TMA. Treatment consisted of glucocorticoids (pulse of intravenous methylprednisolone followed by prednisone tapering), colchicine, fluid drainage, and ultimately pericardiectomy. She did not develop any signs of veno-occlusive disease or acute GVHD despite the discontinuation of cyclosporine 11 months after HSCT.

Two years after HSCT, the patient had a favorable outcome with significant reduction in urine porphyrins levels (although still remaining above the normal range) (Figure 2). Physical examination showed erythrodontia (Figure 1C) without any skin lesions or hepatosplenomegaly. A complete blood count showed a normalization of red cell and platelet counts. Echocardiography, electrolytes, renal function, albumin, and hepatic function were normal.

## Discussion

Several case reports have already proved the effectiveness of HSCT in the treatment of severe CEP.^10–18^ However, our case report presents the impact of a very early diagnosis and management on the outcome of CEP. Our patient suffered from a severe form of CEP due to a homozygous mutation in the UROS gene p. Cys73Arg, the most frequent mutation of CEP. This mutation is also observed in the most severe form of CEP resulting in life-threatening neonatal manifestations or even *in utero* death.^19^ Therefore, our patient underwent an unrelated HSCT from an HLA-matched donor at age 3 months. Hematopoietic stem cell transplantation significantly improved levels of urinary porphyrins although they still remain above the normal range. It suggests HSCT might be used early on in patients with a very severe phenotype of CEP, before the occurrence of any irreversible complications.

In 1991, Kauffman et al^20^ first described encouraging results after a bone marrow graft from a human leucocyte antigen (HLA) identical sibling in a 10-year-old girl with CEP, who eventually died of infectious complications. Early clinical recovery of skin lesions and an important diminution of urinary porphyrin excretion, although slightly above the normal range, were observed 9 months after transplantation, revealing for the first time the possible benefit of HSCT in CEP. Since, more than 25 successful cases have been described.^10–18^ A recent study presented 13 cases of HSCT in CEP, where 11 were still in remission 7 years after transplantation, and 2 died of infectious complications following HSCT.^19^ Besnard et al^21^ recently described the long-term complications post-HSCT of 6 patients during a maximum follow-up period of 25 years. Indeed, 4 patients had a favorable outcome with near-normal levels of porphyrins and no clinical manifestations of CEP while 1 died from severe GVHD and 1 from acute hepatic failure 1 year after HSCT.

Although allogeneic HSCT is known to be a curative treatment of CEP, the decision must be balanced with the risks of complications related to bone marrow transplantation.^22^ Accordingly, HSCT is currently reserved for individuals with severe forms of CEP. Currently, there is a lack of definitive criteria to identify the subgroup of patients suffering from a severe form of CEP, and hence a poor prognosis, who could benefit from HSCT. Moreover, the genotype-phenotype association lacks consistency, as patients with identical genotype may have various clinical presentations.^4,23^ A recent publication proposed that patients with onset of hemolytic anemia or thrombocytopenia by the age of 5 years old have a poorer prognosis,^4^ suggesting that HSCT is indicated.

Early diagnosis is critical, permitting initiation of supportive care before the apparition of irreversible changes. Unfortunately, CEP can be difficult to diagnose with a reported median age of diagnosis of 7 months with typical red urine, CEP skin lesions and hemolytic anemia appearing at median age of 1, 2, and 8 months, respectively.^21^ Martinez Perinado et al^12^ reported an early onset case of CEP who presented with severe post-delivery manifestations and underwent a matched unrelated HSCT at age 7 months. As CEP is a rare condition, a multidisciplinary evaluation led by the responsible pediatrician is necessary for early diagnosis and genetic testing. For our patient, genetic testing before 72 h hours of life allowed the initiation of supportive care and ultimately HSCT.

Urinary porphyrin levels of our patient significantly improved but remain above the normal range. Several case reports of successful HSCT in the treatment of CEP also described a similar situation, notably a recent study describing near-normal biological levels of porphyrins 25 years after HSCT.^21^ The remaining elevated porphyrin levels, despite HSCT, might reflect the porphyrin production by nonerythroid tissues. Indeed, UROS deficiency is still present in nonerythroid tissues (i.e., liver cells) leading to a persistent accumulation of porphyrins despite full replacement of erythroid tissues with HSCT.^2^ The safe threshold of urinary porphyrins for withdrawal of preventive measures remains unknown.

Hematopoietic stem cell transplantation is an effective treatment of CEP conditional to the availability of matched donors. New experimental gene therapies are being studied to overcome this limitation. de Verneuil et al^24^ recently published about genetically modified hematopoietic stem cells, with a lentivirus-mediated vector, allowing the normal expression of the UROS gene to proceed to a bone marrow transplantation in a murine model of CEP. Results showed a complete enzymatic and phenotypic resolution of the disease, suggesting a promising scope for genetically modified HSCT.

To our knowledge, this case report describes the short-term benefits of HSCT in the youngest patient suffering from a severe neonatal phenotype of CEP. It suggests that early diagnosis and treatment might prevent the occurrence of devastating and irreversible consequences of CEP.

## Author Contributions

All authors contributed to the writing of the manuscript and approved the revised version.
